# Rank ectopic expression in the presence of Neu and PyMT oncogenes alters mammary epithelial cell populations and their tumorigenic potential

**DOI:** 10.1007/s10911-023-09530-4

**Published:** 2023-02-18

**Authors:** Alex Cordero, Patricia G. Santamaría, Eva González-Suárez

**Affiliations:** 1 Oncobell, Bellvitge Biomedical Research Institute, IDIBELL, 08908 Barcelona, Spain; 2 Department of Neurological Surgery, Northwestern University Feinberg School of Medicine, Chicago, IL 60611 USA; 3 Molecular Oncology, Spanish National Cancer Research Centre (CNIO), 28029 Madrid, Spain

**Keywords:** Rank, PyMT, Neu, Mammary epithelial cell, Cancer stem cell, Cell of origin, Tumor initiating cell, Metastasis

## Abstract

Determination of the mammary epithelial cell that serves as the cell of origin for breast cancer is key to understand tumor heterogeneity and clinical management. In this study, we aimed to decipher whether Rank expression in the presence of PyMT and Neu oncogenes might affect the cell of origin of mammary gland tumors. We observed that Rank expression in PyMT^+/−^ and Neu^+/−^ mammary glands alters the basal and luminal mammary cell populations already in preneoplasic tissue, which may interfere with the tumor cell of origin restricting their tumorigenesis ability upon transplantation assays. In spite of this, Rank expression eventually promotes tumor aggressiveness once tumorigenesis is established.

**Supplementary Information:**

The online version contains supplementary material available at 10.1007/s10911-023-09530-4.

## Introduction

Rank and its ligand Rankl are key regulators of mammary gland development, controlling the proliferation and differentiation of the mammary epithelium [1, 2]. Rankl is expressed in progesterone receptor positive mammary epithelial cells (MECs) and mediates the proliferative effects of progesterone in mice [1, 3] and human mammary epithelium [4]. In fact, Rank deletion or overexpression results in defective mammary gland development during pregnancy and impaired lactation [1, 2, 5]. We have previously demonstrated that Rank overexpression in the mammary gland, under the mouse mammary tumor virus (MMTV) promoter (Rank^+/tg^), impairs the differentiation of MECs, resulting in an accumulation of mammary stem cells (MaSCs) and intermediate progenitor cells [6]. Rank overexpression also expands both basal (CD24^lo^ CD29^hi^ CD49f^hi^) and luminal (CD24^hi^ CD29^lo^ CD49f^lo^) mammary cell populations and decreases Sca1 and CD61 expression [6], described as markers of luminal differentiated cells and alveolar precursors, respectively [7–10]. Moreover, Rank increases CD49b expression, a marker of luminal progenitor cells, within the luminal population [9].

Rank overexpressing mice spontaneously develop mammary adenocarcinomas containing bipotent progenitor cells, double positive for CK14 and CK8 [6], and show a shorter tumor latency and increased incidence of mammary tumors after carcinogenic protocols compared to controls [11, 12]. Conversely, genetic loss or pharmacological inhibition of Rank prevents or attenuates mammary tumor formation driven by carcinogens [11, 12].

MMTV-Neu (Neu^+/−^) and MMTV-PyMT (PyMT^+/−^) mice develop multifocal luminal-like mammary gland adenocarcinomas with high lung metastatic burden [13–16]. Rank protein is expressed focally in Neu and PyMT non-transformed mammary glands and at higher levels in mammary hyperplasias and invasive adenocarcinomas [11, 17]. The deletion or pharmacological inhibition of Rank signaling in PyMT^+/−^ and Neu^+/−^ models impairs tumor onset and progression [11, 17].

We have recently shown that Rank overexpression in these oncogene-driven mouse models unexpectedly delayed tumor initiation, while promoting tumor aggressiveness [18]. Indeed, Rank overexpression in normal healthy mammary epithelium induces senescence in MECs. Importantly, this Rank-induced senescence is essential for Rank-driven stemness in MECs and breast tumors, preventing tumor initiation but priming cancer stemness and metastatic potential [18].

In this study, we report that Rank expression in the presence of PyMT and Neu oncogenes leads to changes in mammary gland populations which might alter tumor initiation and progression.

## Results

### Rank expression alters mammary epithelial cell populations in Neu and PyMT mouse models

**Fig. 1:**
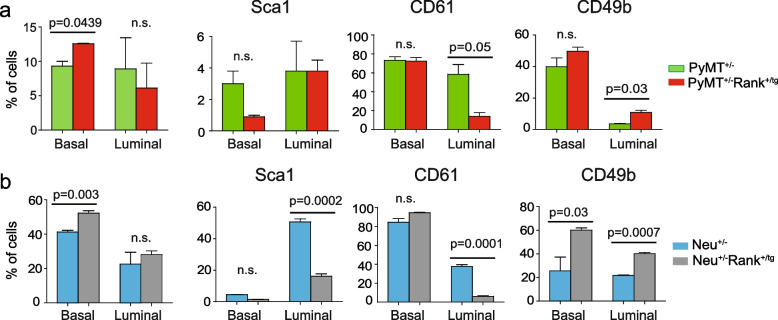
Constitutive Rank expression alters mammary gland populations in PyMT^+/−^and Neu^+/−^ models. **a** and **b** The graphs depict the percentage of basal (CD24^lo^ CD49f^hi^) and luminal (CD24^hi^ CD49f^lo^) cells in the CD45^−^ CD31^−^ Lineage negative (Lin^−^) population and frequency of Sca1^+^, CD61^+^ and CD49b^+^ cells within the basal and luminal populations of PyMT^+/−^ and PyMT^+/−^Rank^+/tg^ mice (**a**) or Neu^+/−^ and Neu^+/−^Rank^+/tg^ mice (**b**) calculated after flow cytometry analyses. Mean, SEM and t-test *p* values for *n* = 3 mice per genotype are shown. n.s.: not significant

We have previously demonstrated that Rank ectopic expression leads to changes in both basal and luminal mammary epithelial subpopulations [6]. Moreover, we found that Rank induced a significant delay in the appearance of mammary gland tumors and a reduced tumor incidence in PyMT^+/−^Rank^+/tg^ and Neu^+/−^Rank^+/tg^ mouse models [18]. Aiming to understand whether Rank, in cooperation with PyMT and Neu oncogenes, influenced mammary epithelial cell distribution resulting in a delayed tumor onset in double transgenic mice, we analyzed by flow cytometry the mammary cell populations in PyMT^+/−^Rank^+/tg^ and Neu^+/−^Rank^+/tg^ mice before palpable lesions were detected. As we previously found that the MMTV promoter drives *Rank* expression to both luminal and basal cells in the Rank^+/tg^ mice, we reasoned that *Rank* would be similarly expressed in those populations in the double transgenic mice [6]. In PyMT^+/−^ (FVB), the first palpable tumors appeared at puberty [16]. For this reason, we performed an analysis of pre-pubertal mammary glands. Analyses by flow cytometry of 2.2–2.6-week-old mice showed an increase in CD24^lo^ CD49f^hi^ basal cells and CD49b^+^ luminal cells and a decrease in CD61^+^ luminal cells in PyMT^+/−^Rank^+/tg^ compared to PyMT^±^ mammary glands (Fig. 1a). Sca1^+^ cells were few in PyMT^+/−^Rank^+/tg^ and PyMT^+/−^ mice, in accordance with the low expression of Sca1 at this age [10, 19]. In Neu^+/−^Rank^+/tg^ mice, preneoplastic virgin mammary glands from 23–25-week-old females showed the same alterations in mammary cell populations previously described for Rank^+/tg^ mice [6], with increased basal (CD24^lo^ CD49f^hi^) and luminal (CD24^hi^ CD49f^lo^) subpopulations, less Sca1^+^ and CD61^+^ luminal cells, and more CD49b^+^ cells, as compared to single Neu^+/−^ mammary glands (Fig. 1b). These findings confirmed that Rank expression in the PyMT^+/−^ and Neu^+/−^ mouse models disrupts the distribution of mammary populations in virgin mammary glands similarly than in Rank^+/tg^ mice [6].

### PyMT^+/−^ MECs drive luminal tumor formation regardless of the cell of origin and Rank expression levels

**Fig. 2:**
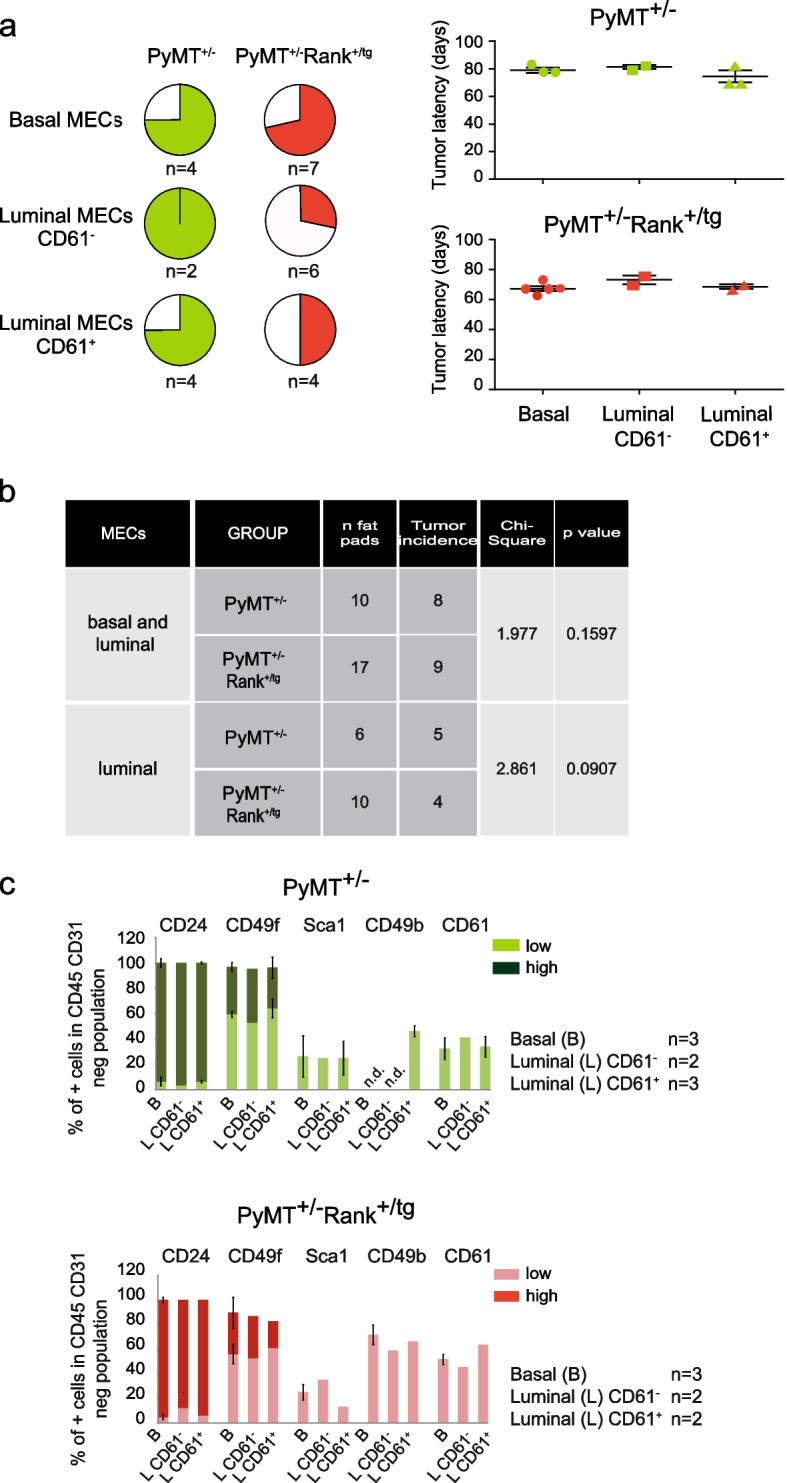
Tumor formation by basal and luminal MECs isolated from PyMT^+/−^Rank^+/tg^ mice. **a** Tumor incidence (pie charts on the left) and tumor latency (graphs on the right) observed in mice injected with Lin^−^ basal (CD24^lo^ CD49f^hi^), CD61^−^ luminal (CD24^hi^ CD49f^lo^ CD61^−^) and CD61^+^ luminal (CD24^hi^ CD49f^lo^ CD61^+^) MECs from 2.2–2.6-week-old PyMT^+/−^ and PyMT^+/−^ Rank^+/tg^ mice. Two thousand cells from each sorted population were injected/gland. The number of injected mammary glands with each cell population is indicated below the corresponding pie chart. **b** Contingency table analysis of tumor formation from basal and luminal PyMT^+/−^ and PyMT^+/−^Rank^+/tg^ MECs. **c** Tumors generated as depicted in (**a**) were analyzed for the frequency of CD24^hi/lo^, CD49f^hi/lo^, Sca1^+^, CD49b^+^ and CD61^+^ cell populations in the Lin^−^ population. Positive/negative and high(hi)/low(lo) populations were determined according to populations in the normal mammary gland. Note that only 3 out of 5 tumors that grew upon basal PyMT^+/−^ Rank^+/tg^ MEC injection were analyzed. Mean, SEM (when *n* > 2) and number of tumors analyzed for each condition is shown. n.d.: no data was obtained due to insufficient cells

Since PyMT^+/−^ mice developed luminal CK8^+^ tumors with enhanced *Rank* expression compared with non-tumorigenic mammary epithelia adenocarcinomas [11, 17] and Rank overexpression led to a reduction in the CD61^+^ luminal populations [18], we hypothesized that tumors might originate from these luminal populations, explaining the attenuation of tumorigenesis observed in PyMT^+/−^Rank^+/tg^ double transgenic mice [18]. Therefore, we isolated basal (Lin^−^ CD24^lo^ CD49f^hi^) and luminal CD61^+^ and CD61^−^ (Lin^−^ CD24^hi^ CD49f^lo^ CD61^±^) cells from 2.2–2.6-week-old PyMT^+/−^ and PyMT^+/−^Rank^+/tg^ mice (before clonal tumor populations were detected) and implanted them orthotopically into the mammary fat pads of syngeneic FVB female mice. Every PyMT^+/−^ and PyMT^+/−^Rank^+/tg^ derived cell population gave rise to lesions that showed a similar latency, however, MECs from PyMT^+/−^Rank^+/tg^ formed less tumors than those from single transgenic PyMT^+/−^ mice, although the limited number of grown tumors prevented finding significant differences (Fig. 2a, b). We next analyzed by flow cytometry tumors originated upon PyMT^+/−^Rank^+/tg^ basal and luminal MEC injection and a slight increase in CD49b and CD61 cell populations was detected when compared to PyMT^+/−^ derived tumors (Fig. 2c), as seen in PyMT^+/−^ and PyMT^+/−^Rank^+/tg^ primary tumors [18]. These results indicate that Rank expression limits to some extent the tumor initiation ability of PyMT MECs, which in turn would explain the reduced tumor burden seen in double transgenic PyMT^+/−^Rank^+/tg^ compared to single PyMT^+/−^ mice [18].

###  Rank overexpression impairs tumor initiation by Neu^+/−^ basal and luminal MECs

**Fig. 3:**
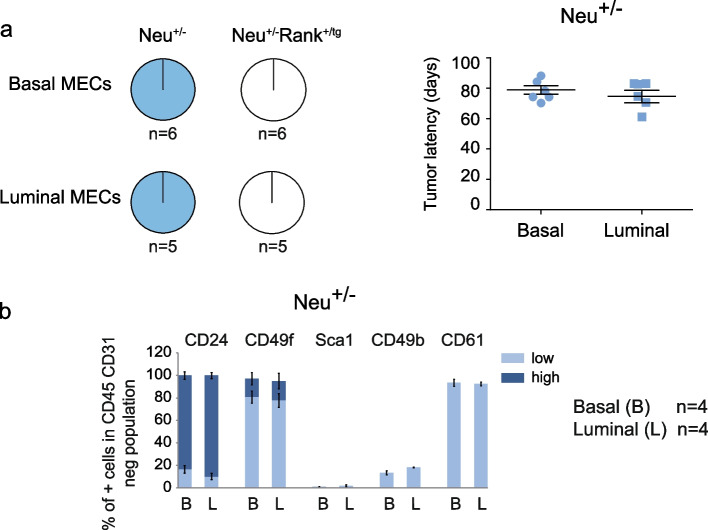
Tumor formation by basal and luminal MECs isolated from Neu^+/−^Rank^+/tg^ mice. **a** Tumor incidence (pie charts on the left) and tumor latency (graphs on the right) in mice injected with Lin^−^ basal (CD24^lo^ CD49f^hi^) or luminal (CD24^hi^ CD49f^lo^) MECs isolated from 20–25-week-old virgin Neu^+/−^ and Neu^+/−^Rank^+/tg^ mice. Fifty thousand cells from each sorted population were injected/gland. The number of independent experiments performed is shown below each pie chart. **b** Tumors generated as depicted in (**a**) were analyzed for the frequency of CD24^hi/lo^, CD49f^hi/lo^, Sca1^+^, CD49b^+^ and CD61^+^ cells in the Lin^−^ population. Positive/negative and high(hi)/low(lo) populations were determined according to populations in the normal mammary gland. Mean, SEM (when *n* > 2) and number of tumors analyzed per group is depicted

Regarding Neu^+/−^ mouse models, Neu^+/−^Rank^+/tg^ mammary glands and tumors expressed significantly higher levels of Rank compared to Neu^+/−^ mice (Fig. S1a) and mice from both genotypes developed luminal-like tumors expressing high levels of CK8 (mRNA and protein), while basal CK5 and CK14 markers were not detected (Fig. S1a, b). Freshly isolated tumor cells were analyzed by flow cytometry revealing that both models developed luminal (CD24^hi^ CD49f^lo^) tumors with high expression of CD61 and low levels of Sca1 and CD49b (Fig. S1c). To understand the delayed tumor onset seen in double Neu^+/−^Rank^+/tg^ compared to single Neu^+/−^ mice [18], basal (Lin^−^ CD24^lo^ CD49f^hi^) and luminal (Lin^−^ CD24^hi^ CD49f^lo^) MECs isolated from non-transformed Neu^+/−^ and Neu^+/−^Rank^+/tg^ mammary glands (23–25-week-old mice) were orthotopically implanted in immunocompromised mice. All mice injected with basal and luminal Neu^+/−^ MECs developed luminal tumors with similar latency and phenotype (CD24^hi^ CD49f^lo^ CD61^+^ CD49b^lo^ Sca1^−^) (Fig. 3a, b). Strikingly, none of the mice injected with basal or luminal Neu^+/−^Rank^+/tg^ MECs developed tumors six months after the injection (Fig. 3a), indicating that Rank overexpression drastically diminishes the tumor forming ability of luminal and basal MECs derived from the Neu^+/−^ background.

### Rank overexpression enhances tumor aggressiveness

**Fig. 4:**
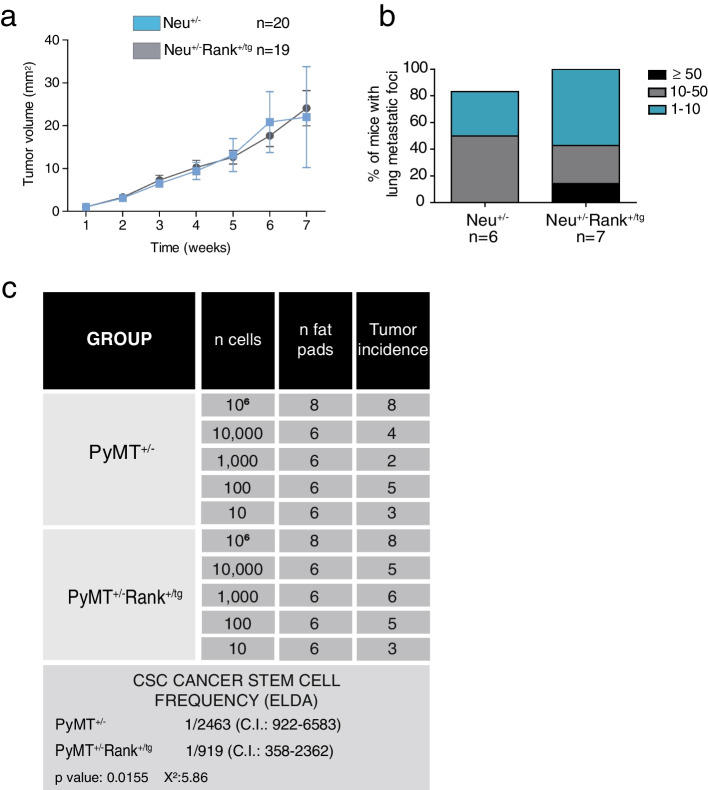
Rank ectopic expression enhances aggressiveness in the presence of Neu and PyMT oncogenes. **a** Relative tumor growth in Neu^+/−^ and Neu^+/−^Rank^+/tg^ mice. Mean tumor volume ± SEM at each time point relative to their volume on the first day of detection is shown. **b** Percentage of mice with the indicated number of lung metastatic foci is shown. The number of mice analyzed from each genotype is indicated. **c** Table showing the results obtained from a limiting dilution assay to test the tumor initiating ability of PyMT^+/−^ and PyMT^+/−^Rank^+/tg^ tumor cells. Two independent PyMT^+/−^ and PyMT^+/−^Rank^+/tg^ tumors were pooled and the indicated number of cells were injected into the abdominal mammary glands from FBV wt females. The tumor initiating cell frequencies with confidence intervals (C.I.) for each group were calculated by ELDA [35]; p and chi-square (χ^2^) values are shown

We previously showed that tumors in PyMT^+/−^Rank^+/tg^ mice grew faster and seeded more metastasis than those from single PyMT^+/−^ mice, despite their delayed tumor onset [18]. In the Neu background, no clear changes in tumor growth were observed between Neu^+/−^ and Neu^+/−^Rank^+/tg^ mice once tumors were established (Fig. 4a), but the lung metastatic burden was higher in Rank overexpressing Neu^+/−^ mice (Fig. 4b). These differences may be related to the increase in CD49b^+^ and CD61^+^ progenitor-like cells seen in Rank overexpressing PyMT^+/−^ mice, with an accumulation of bipotent CK14^+^/CK8^+^ cells in established tumors [18], which was not detected neither in Neu^+/−^ nor in Neu^+/−^Rank^+/tg^ mice (Fig. S1b).

Together, these results indicate that the tumor initiating ability of MECs derived from the double transgenic models is either reduced (PyMT^+/−^) or blocked (Neu^+/−^) in the presence of Rank (Figs. 2a and 3a), paradoxically, double transgenic tumors are more aggressive. Since this is particularly compelling in the PyMT^+/−^background, we performed an orthotransplatation limiting dilution assay to estimate the frequency of tumor-initiating cells in breast tumors from PyMT^+/−^ and PyMT^+/−^Rank^+/tg^ mice. This approach estimated a higher frequency of cancer stem cells in PyMT^+/−^Rank^+/tg^ tumor cells than in PyMT^+/−^ mice (Fig. 4c) even though the differences in tumor burden were small. These results support that Rank contributes to tumor cell aggressiveness, predominantly in the PyMT^+/−^ background. Still, Rank increases metastasis seeding ability in both Neu^+/−^ and PyMT^+/−^ tumorigenic settings indicating that Rank favors metastatic colonization independently of primary tumor proliferation.

## Discussion

Identifying the cell of origin in different breast cancer subtypes is key for understanding breast cancer heterogeneity and has relevant therapeutic implications [10, 20, 21]. Recent advances in this area derive from the isolation of stem and progenitor cells and functional assays such as in vivo transplantation experiments, lineage-tracing studies and single-cell RNA sequencing efforts [21–25]. Besides, it is well established that specific oncogenic events in different mammary cell populations lead to dissimilar tumor outcome, as has been shown for the PIK3CA H1047R oncogene that induces distinctive tumors depending on the cellular origin in which the oncogene is expressed [26, 27]. RANK aberrant activation has already been linked to basal-like cancers arising in *BRCA1* mutation carriers, likely being an early event in preneoplasic *BRCA1*^*mut/*+^ breast tissue that favours oncogenesis [28].

In the current manuscript, we reveal that Rank ectopic expression in the mouse mammary gland influences mammary cell fate already in preneoplasic glands from oncogene-driven mouse models. We demonstrate that, before tumorigenesis is established, Rank is sufficient to alter the distribution of basal and luminal mammary cell subpopulations, reducing tumorigenic potential upon transplantation.

In Neu^+/−^ and PyMT^+/−^ tumor models, the cancer cell of origin remains controversial [29–31]. We aimed to understand in what manner Rank expression influences the distribution of mammary epithelial populations and the tumor initiating ability of Neu^+/−^ and PyMT^+/−^ derived MECs. In a recent manuscript, we demonstrated that Rank ectopic expression in the mammary epithelia delays tumor onset and reduces tumor incidence in the Neu^+/−^ and PyMT^+/−^ models by inducing cell senescence. Paradoxically, Rank promotes tumor aggressiveness by favouring basal and luminal cell stemness [18]. We thus interrogated MECs from single and double transgenic mice to understand how Rank, in the presence of Neu and PyMT oncogenes, influences mammary epithelial hierarchy and ultimately affects tumorigenesis.

Here we show that Rank expression in PyMT^+/−^ and Neu^+/−^ mammary glands induced similar alterations in basal and luminal mammary cell populations than previously described in Rank^+/tg^ mice, where we demonstrated that *Rank* was expressed in basal and luminal compartments [6]. This indicates that PyMT and Neu oncogenes do not contribute any further to changes in MEC distribution, at least in preneoplasic mammary glands. The expression of Rank in PyMT^+/−^ and Neu^+/−^ mammary glands decreased the frequency of luminal progenitor subpopulations, identified by Sca1 and CD61 marker expression, however, Rank restrained luminal as well as basal MEC tumor initiation potential in both backgrounds. Orthotopic injection of basal and luminal MECs from Neu^+/−^ and PyMT^+/−^ models led, in both cases, to luminal tumor formation. In the mouse mammary glands, the MMTV promoter drives the expression of these oncogenes mostly within the luminal epithelial compartment [32], therefore, we expected that Neu and PyMT oncogenes mediated the transformation of luminal cells resulting in tumors that lose the myoepithelial cell population [21, 27]. In our experimental setting, basal MECs also gave rise to luminal tumors, which could stem from the activation of the MMTV promoter within distinct cell types [33] or from a previous step, in which basal cells differentiate into luminal cells upon transplantation giving then rise to luminal tumors. Remarkably, Rank concomitant expression prevented the tumor initiation ability of luminal and basal Neu^+/−^ MECs, whereas in PyMT^+/−^ mice, Rank expression reduced to some degree basal and luminal MEC tumor initiation potential. Therefore, Rank expression before tumor establishment hinders tumor formation independently of the tumor cell of origin, which may explain the delayed tumor onset seen in PyMT^+/−^Rank^+/tg^ compared to single PyMT^+/−^mice [18]. Unlike primary tumors, tumor latency was not altered upon transplantation assays of MECs derived from pre-pubertal mammary glands from PyMT^+/−^Rank^+/tg^ compared to single PyMT^+/−^ mice. This is likely due to the fact that Rank-induced senescent cells would be negatively selected during cell isolation. Besides, the Rank protective effect on Neu^+/−^Rank^+/tg^ double transgenic mice with delayed tumor latency and decreased tumor frequency than their Neu^+/−^ counterparts [18] was even greater upon orthotopic transplants, as neither basal nor luminal Neu^+/−^Rank^+/tg^ MECs gave rise to tumors, not even in a fully immunocompromised environment.

However, once tumors are established, PyMT^+/−^Rank^+/tg^ tumor cells showed fairly increased tumor initiating ability compared to single PyMT^+/−^ tumors, in line with their enhanced stemness and metastatic potential, probably due to the accumulation of luminal progenitor populations CD61^+^ and CD49b^+^ and embryonic-like dual positive CK14/CK8 cells [18]. In Neu^+/−^Rank^+/tg^ established tumors, no bipotent progenitor cells were detected, still, Rank expression enhanced tumor aggressiveness as determined by the increased lung metastatic colonization ability compared to single Neu^+/−^ mice.

Finally,﻿ we cannot discard that additional mechanisms driven by Rank (not only senescence and alterations in the mammary epithelium populations), combined with the specific and distinctive effects derived from the PyMT and Neu oncogenes, can contribute to the observed differences in tumor initiation and progression between single and double transgenic mice.

Building on our previous results showing that Rank-induced senescence was responsible for aggressiveness once tumorigenesis was established [18], we here demonstrate that, up to that point, perturbation in Rank expression hinders the potential of tumorigenic-prone cells to originate tumors by altering the distribution of mammary epithelial populations. Our results also indicate that PyMT and Neu oncogenes under the MMTV promoter have a deterministic role on the cell of origin since basal and luminal MECs from single transgenic mice give rise to luminal tumors independently of Rank expression. Although further experiments are required to comprehend how Rank enhances the metastatic dissemination of MECs in the context of PyMT and Neu oncogenes, we believe that the present results together with the extensive data on PyMT^+/−^Rank^+/tg^ mice shown in our previous work [18], provide relevant insight into the consequences of aberrant Rank expression in an oncogenic background. These and previous reports [28] highlight the need to fully understand RANK signaling in different breast cancer oncogenic-contexts in order to make better use of RANK targeting therapies.

## Methods

### In vivo animal studies

All research involving animals was performed at the IDIBELL animal facility in compliance with protocols approved by the IDIBELL Committee on Animal Care and following national and European Union regulations. Mouse models used in this study have been previously described: MMTV-Rank^+/tg^ (Rank^+/tg^) [2, 5], MMTV-Neu (Neu^+/−^) [14], MMTV-PyMT (PyMT^+/−^) [13] and double transgenic PyMT^+/−^Rank^+/tg^ and Neu^+/−^Rank^+/tg^ [18]. Mice were monitored for tumor formation three times per week and animals bearing tumors bigger than 1 cm in diameter were considered as endpoint criteria for sacrifice.

### MECs and mammary tumor cell isolation

Single cells were isolated from mammary glands or tumors as previously described [18, 34]. Briefly, fresh tissues were mechanically dissected with McIlwain tissue chopper and enzymatically digested with DMEM/F12 (Gibco), 0.3% collagenase A (Sigma), 2.5 U/ml dispase (Sigma), 20 mM HEPES and Penicillin/Streptomycin (ThermoFisher Scientific) for 45 min at 37 °C. For MEC isolation, fibroblasts were excluded by incubation with DMEM high glucose containing 10% FBS (Gibco) for 1 h at 37 °C. Single MECs were then isolated by trypsin (Gibco) incubation for 2 min at 37 °C followed by an incubation with 2.5 U/ml dispase I, 20 U/ml DNase I (Roche) for 5 min at 37 °C. Cell aggregates were removed by filtering the cell suspensions with 40 μm cell strainers (BD Falcon). For tumor cell isolation, samples were treated with trypsin for 2 min at 37°C and cell aggregates were removed by filtering the cell suspension with 70 μm strainers (BD Falcon).

### Orthotransplantation assays

For orthotopic transplants, basal (Lin^−^ CD24^lo^ CD49f^hi^) and luminal (Lin^−^ CD24^hi^ CD49f^lo^) MECs isolated from mammary glands as detailed above were diluted 1:1 in matrigel matrix (Culteck) and injected in a final volume of 40 µL in the abdominal mammary fat pad of immunocompromised female mice (50,000 cells/gland from Neu^+/−^ and Neu^+/−^Rank^+/tg^ mice) or syngeneic females (2,000 cells/gland from PyMT^+/−^ and PyMT^+/−^Rank^+/tg^). Mice were monitored for tumor incidence and latency and sacrificed when tumors reached a volume of 1 cm^3^.

### Tumor limiting dilution assay

Mammary tumor cells from PyMT^+/−^ and PyMT^+/−^Rank^+/tg^ primary tumors were isolated as described above, pooled, diluted 1:1 in matrigel matrix (Culteck) and injected in a final volume of 40 µL. 1.000,000, 10,000, 1,000, 100 and 10 cells were injected in the abdominal mammary fat pads of 8-week-old syngeneic mice (FVB background) and tumor incidence was monitored along time.

### Flow cytometry

Single cells were labeled with antibodies against CD24-PE or CD24-FITC (5 μg/mL, M1/69 BD Pharmingen, San Diego, CA, http://www.bdbiosciences.com), CD29-FITC (1.25 μg/mL, HMb1-1, BD Pharmingen), CD49f-a647 (2.5 μg/mL, GoH3, BD Pharmingen), CD61-PE or CD61-FITC (2.5 μg/mL, 2C9.G2, BD Pharmingen), Sca1-APC or Sca1-PE (0.5 μg/mL, Ly-6A/E, BD Pharmingen), and CD49b-a647 (1.25 μg/mL, HMa2 Biolegend, San Diego, CA, http://www.biolegend.com). Lymphocytes and endothelial cells were excluded in flow cytometry using CD45-PECy7 (0,125 μg/mL, 30-F11 Biolegend) and CD31-PECy7 (0,5 μg/mL, 390 Biolegend) antibodies, respectively. Flow cytometry analysis was performed using FACS Canto (Becton Dickinson, San Jose, CA) and Diva software package. Cell sorting was performed using MoFlo (Beckman Coulter) at 25 psi and using a 100 mm tip.

### Tissue histology and immunostaining

Tissue samples were fixed in formalin and embedded in paraffin and 3–5 μm sections were cut for histological analyses and stained with hematoxylin and eosin. Lung metastases were detected and counted based on nuclear morphology and similarity with primary tumors. 15–16 sections per lung were quantified in primary Neu^+/−^Rank^+/tg^ and Neu^+/−^ tumors. Immunostaining was performed on 3 μm tumor sections. After citrate antigen heat retrieval, slides were stained with CK8 (TROMA, dshl, Developmental Studies Hybridoma Bank, Iowa City, Iowa), CK5 (AF-138, Covance, Princeton, NJ) and CK14 (AF-64, Covance) antibodies followed by incubation with corresponding fluorochrome conjugated secondary antibodies (Life Technlogies) and DAPI (Sigma). Slides were mounted with Prolong ® Gold Antifade (Life Technologies) and analyses were carried out in a Leica TCS SP5 confocal microscope. Images were captured using Las AF Lite software (Leica).

### Gene expression analysis

Total RNA of frozen tumor pieces was prepared with Tripure Isolation Reagent (Roche) following the manufacturer’s instructions. Frozen tumors tissues were fractionated using glass beads (Sigma) and PreCellys® tissue homogenizer (Berting Technologies). cDNA was produced by reverse transcription using 1 μg of RNA following kit instructions (Applied Biosystems). Quantitative PCR was performed using LightCycler® 480 SYBR green MasterMix (Roche) and the primers used were already published [18].

### Statistical analysis

Statistical analyses were performed using GraphPad Prism software. Analysis of the differences between mouse cohorts or conditions was performed with a two-tailed Student’s t-test, one-way ANOVA or Chi-Square test. *p* values are annotated; n.s. not significant. Estimation of tumor-initiating cells in limiting dilutions was calculated using the extreme limiting dilution analysis (ELDA) [35].

## Supplementary Information

**Additional file 1: Supplementary Fig. 1.** Tumors formed in Neu^+/-^ and Neu^+/-^Rank^+/tg^ mice show a luminal-like phenotype. **a** mRNA expression of *Rank,* *Krt8 and Krt14* relative to *Pp1a* measured by qPCR in pretumoral mammary glands (MG) and tumors (T) from Neu^+/-^ and Neu^+/-^Rank^+/tg^ mice. Mean, SEM and t-test p value for indicated mammary glands and tumors are shown. Quantifications were performed in triplicate and mean values were used in the calculations. **b** Representative CK8 (shown in red) and CK14/CK5 (in green) immunostainings in Neu^+/-^ and Neu^+/-^Rank^+/tg^ spontaneous tumor lesions. **c** Frequency of CD24^hi/lo^, CD49f^hi/lo^, Sca1^+^, CD49b^+^and CD61^+^ cells in CD45^-^ CD31^-^ Lin^-^ population determined in Neu^+/-^ and Neu^+/-^Rank^+/tg^ spontaneous tumors analyzed by flow cytometry. Positive/negative and high(hi)/low(lo) populations were set according to populations in the normal mammary gland. Mean and SEM for the indicated number of tumors from each genotype are shown.
