# Correction to: Rank ectopic expression in the presence of Neu and PyMT oncogenes alters mammary epithelial cell populations and their tumorigenic potential

**DOI:** 10.1007/s10911-023-09532-2

**Published:** 2023-03-07

**Authors:** Alex Cordero, Patricia G. Santamaría, Eva González‑Suárez

**Affiliations:** 1 Oncobell, Bellvitge Biomedical Research Institute, IDIBELL, 08908 Barcelona, Spain; 2 Department of Neurological Surgery, Northwestern University Feinberg School of Medicine, Chicago, IL 60611 USA; 3 Molecular Oncology, Spanish National Cancer Research Centre (CNIO), 28029 Madrid, Spain

**Correction to: Journal of Mammary Gland Biology and Neoplasia 28, 2 (2023)**

10.1007/s10911-023-09530-4

Following publication of the original article [1], the author requested to correct the word “MMTV“ to “PyMT“ in the article title. The correct article title should be:

“Rank ectopic expression in the presence of Neu and PyMT oncogenes alters mammary epithelial cell populations and their tumorigenic potential”

The original article [1] has been corrected.
