# Supplementary figures and images for: Rank ectopic expression in the presence of Neu and PyMT oncogenes alters mammary epithelial cell populations and their tumorigenic potential
Source: J Mammary Gland Biol Neoplasia. 2023 Feb 18;28(1):2. doi: 10.1007/s10911-023-09530-4 (PMC9938814; doi:10.1007/s10911-023-09530-4)

Figure S1

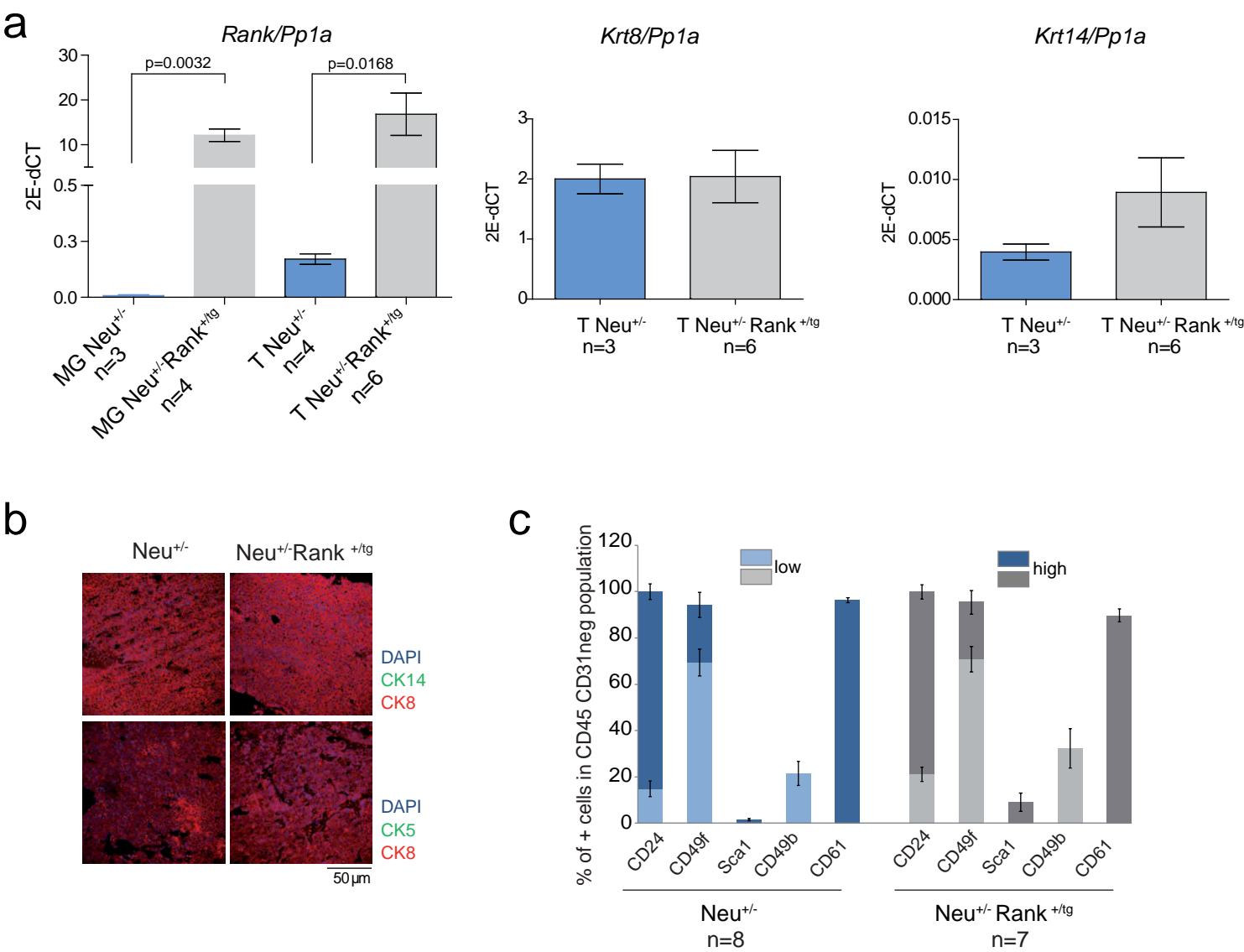

Supplement: Supplementary file 1 — Additional file 1: Supplementary Fig. 1. Tumors formed in Neu+/- and Neu+/-Rank+/tg mice show a luminal-like phenotype. a mRNA expression of Rank, Krt8 and Krt14 relative to Pp1a measured by qPCR in pretumoral mammary glands (MG) and tumors (T) from Neu+/- and Neu+/-Rank+/tg mice. Mean, SEM and t-test p value for indicated mammary glands and tumors are shown. Quantifications were performed in triplicate and mean values were used in the calculations. b Representative CK8 (shown in red) and CK14/CK5 (in green) immunostainings in Neu+/- and Neu+/-Rank+/tg spontaneous tumor lesions. c Frequency of CD24hi/lo, CD49fhi/lo, Sca1+, CD49b+and CD61+ cells in CD45- CD31- Lin- population determined in Neu+/- and Neu+/-Rank+/tg spontaneous tumors analyzed by flow cytometry. Positive/negative and high(hi)/low(lo) populations were set according to populations in the normal mammary gland. Mean and SEM for the indicated number of tumors from each genotype are shown. [file 10911_2023_9530_MOESM1_ESM.pdf]
